# Safety and efficacy of perioperative FLOT regimen in Japanese patients with gastric, esophagogastric junction, or esophageal adenocarcinoma: a single-institution experience

**DOI:** 10.1016/j.esmogo.2024.100050

**Published:** 2024-04-17

**Authors:** S. Takei, A. Kawazoe, A. Jubashi, M. Komatsu, K. Sato, S. Mishima, D. Kotani, M. Yura, N. Sakamoto, S. Sakashita, T. Kuwata, T. Kojima, T. Fujita, T. Kinoshita, K. Shitara

**Affiliations:** 1 Department of Gastroenterology and Gastrointestinal Oncology; 2 Department of Gastric Surgery; 3 Department of Esophageal Surgery; 4 Department of Pathology and Clinical Laboratories; 5 Department of Genetic Medicine and Services, National Cancer Center Hospital East, Kashiwa, Chiba; 6 Department of Immunology, Nagoya University Graduate School of Medicine, Nagoya, Aichi, Japan

**Keywords:** esophageal adenocarcinoma, FLOT, gastric cancer, gastroesophageal junction cancer, Japanese patients, perioperative chemotherapy

## Abstract

**Background:**

Although the common treatment strategy for localized gastric cancer in Japan is gastrectomy followed by adjuvant chemotherapy, several randomized studies in non-Japanese populations have established perioperative chemotherapy as the standard treatment of localized gastric or gastroesophageal junction adenocarcinoma. Therefore, we have implemented this strategy in our institution.

**Patients and methods:**

We retrospectively reviewed the medical records of patients with resectable gastric cancer, gastroesophageal junction cancer, and esophageal adenocarcinoma who had received perioperative FLOT (5-fluorouracil plus docetaxel plus oxaliplatin plus leucovorin) from February 2020 to February 2023.

**Results:**

In this study, a total of 91 patients were analyzed, with a median age of 70 years (range: 29-82). At the time of diagnosis, 83 patients (91.2%) had T3 or higher-grade primary lesions, and 85 (93.4%) had lymph node metastasis. A total of 10 patients had resection before completing four cycles of preoperative chemotherapy, and 77 of 91 (84.6%) completed four cycles with 74 of them receiving radical resection. Among the 84 patients who had radical resection after FLOT, 82 (97.6%) achieved R0 resection, including 8 (9.5%) with a pathological complete response. After resection, 60 patients (65.9%) received at least one cycle of post-operative FLOT, and 47 (51.6%) completed eight cycles of FLOT treatment. Chemotherapy-related adverse events of grade 3 or higher occurred during the pre- and post-operative FLOT in 60 patients (65.9%), including leukopenia (30.8%), neutropenia (50.5%), febrile neutropenia (5.5%), and anorexia (7.7%). No treatment-related deaths occurred.

**Conclusions:**

These findings were comparable to those in the pivotal FLOT 4 trial, suggesting acceptable feasibility of the FLOT regimen in Japanese clinical practice.

## Highlights

• Safety and efficacy of FLOT in Japanese gastric/junction/esophageal adenocarcinoma.
• Primary prophylactic G-CSF effectively prevent neutropenia and febrile neutropenia.
• Limited efficacy of FLOT in dMMR/MSI-H cases.

## Introduction

Gastric cancer is the fifth most common cancer in the world and the fourth leading cause of cancer-related deaths.^1^ Surgical resection is the only curative treatment option for localized gastric or gastroesophageal junction adenocarcinoma.^2^, ^3^, ^4^, ^5^, ^6^ The recurrence rates following resection remain high, however, and multimodality treatment strategies, such as perioperative (neoadjuvant/adjuvant) chemotherapy, adjuvant chemotherapy, or chemoradiation, are generally recommended for gastric or gastroesophageal junction adenocarcinoma.^3^, ^4^, ^5^, ^6^ In the MAGIC and FNCLCC/FFCD trials, perioperative chemotherapies with triplet or doublet regimens, including fluoropyrimidine and platinum, demonstrated better survival outcomes than surgery alone.^7^^,^^8^ Moreover, the AIO FLOT4 trial in Germany revealed that the perioperative FLOT regimen (5-fluorouracil plus docetaxel plus oxaliplatin plus leucovorin) is superior to the ECF/ECX regimen (epirubicin plus cisplatin plus 5-fluorouracil or capecitabine).^9^ The Neo-AEGIS trial, a phase III study, found no significant difference in efficacy between the CROSS regimen (carboplatin plus paclitaxel and radiotherapy) and perioperative chemotherapy for patients with esophageal and esophagogastric junction adenocarcinoma.^10^ Of note, most of the patients received the modified MAGIC regimen (epirubicin plus cisplatin/oxaliplatin and fluorouracil/capecitabine) rather than FLOT in Neo-AEGIS. Perioperative chemotherapy is now the standard treatment strategy in Europe and the United States for gastric and gastroesophageal junction adenocarcinoma. Furthermore, it is an acceptable treatment option for esophageal adenocarcinoma.^5^ Contrarily, in Asia, post-operative adjuvant chemotherapy is established as a standard treatment after D2 gastrectomy according to phase III trials.^11^, ^12^, ^13^ Recently, two phase III trials conducted in Asia (RESOLVE in China and PRODIGY in Korea) demonstrated that adding neoadjuvant chemotherapies (S-1 plus oxaliplatin in RESOLVE and docetaxel plus oxaliplatin plus S-1 in PRODIGY) to D2 gastrectomy and adjuvant chemotherapy improved in both disease-free survival and overall survival in RESOLVE, and in progression-free survival and overall survival in PRODIGY.^14^, ^15^, ^16^, ^17^ In Japan, a phase III trial of neoadjuvant S-1 plus oxaliplatin (JCOG1509) is currently ongoing.^18^ Furthermore, the addition of immune checkpoint inhibitors (ICIs) to cytotoxic chemotherapy in the perioperative setting is being investigated in global randomized phase III trials such as KEYNOTE-585 (pembrolizumab plus cisplatin-based chemotherapy or FLOT), MATTERHORN (durvalumab plus FLOT), and DANTE (atezolizumab plus FLOT). In KEYNOTE-585, the pembrolizumab combination group showed a higher rate of pathological complete response (pCR), although there was no statistically significant difference in event-free survival (EFS).^19^ Similarly, MATTERHORN and the phase II portion of DANTE have shown an improvement in pCR rates with the combination of ICI.^20^^,^^21^ The analysis of EFS, the primary endpoint, from these studies is anticipated.

Overall, based on the aforementioned randomized studies, the use of perioperative chemotherapy has consistently demonstrated a clinically meaningful efficacy in the treatment of localized gastric cancer. Therefore, we have attempted to implement this treatment strategy in our institution. In this study, we retrospectively investigated the safety and efficacy of the perioperative FLOT regimen in Japanese patients with localized gastric cancer, gastroesophageal junction cancer, and esophageal adenocarcinoma treated at our institution.

## Materials and methods

### Patients

This retrospective observational study was designed to evaluate the safety and efficacy of perioperative chemotherapy with FLOT in Japanese patients with gastric cancer, gastroesophageal junction cancer, and esophageal adenocarcinoma. We reviewed the medical records of consecutive patients who had received FLOT in a perioperative setting at our institution from February 2020 to February 2023. The eligibility criteria were the presence of histologically proven locally advanced resectable gastric cancer, gastroesophageal junction cancer, and esophageal adenocarcinoma; Eastern Cooperative Oncology Group (ECOG) performance status (PS) of 0-1; adequate bone marrow, hepatic, and renal function; and at least one cycle of preoperative FLOT. Informed consent for chemotherapy was obtained from each patient before the treatment initiation. The study was approved by the ethics committee of our institution and was conducted in accordance with the guidelines for biomedical research stipulated in the Declaration of Helsinki (Approval ID: 2017-120).

### Treatment plan

The FLOT chemotherapy regimen was administered according to the FLOT4 trial.^9^ The patients received the FLOT regimen for four cycles each before and after surgery. Each 2-week cycle consisted of 50 mg/m^2^ docetaxel (i.v.) on day 1, 85 mg/m^2^ oxaliplatin (i.v.) on day 1, 200 mg/m^2^ leucovorin (i.v.) on day 1, and a 24-h infusion of 2600 mg/m^2^ 5-fluorouracil (i.v.) on day 1. In cases of unacceptable toxicity, disease progression, patient death, or upon patient request, treatment was terminated prematurely. The dose reduction of docetaxel, oxaliplatin, or 5-fluorouracil at the start of treatment was decided by each investigator depending on ECOG PS, age, or organ function. Additional dose modification or treatment postponement were decided by each investigator. Transthoracic subtotal esophagectomy with three-field lymphadenectomy or a gastrectomy with D2 lymphadenectomy was carried out based on the consensus of a multidisciplinary team.

### Molecular characteristics

Molecular characteristics, such as the status of the human epidermal growth factor receptor 2 (HER2), programmed death-ligand 1 (PD-L1), and mismatch repair (MMR) deficiency, were analyzed using formalin-fixed paraffin-embedded tissue specimens from archival tissue samples, if available. To evaluate the HER2 status, immunohistochemistry (IHC) using a monoclonal anti-HER2 antibody [PATHWAY HER2 (4B5), Ventana, Tucson, AZ] and fluorescence *in situ* hybridization (FISH) using the PathVysion HER-2 Probe Kit (Abbott Laboratories, Abbott Park, IL) were carried out, and HER2 positivity was defined as IHC 3+ or IHC 2+ and FISH-positive. PD-L1 IHC was carried out using an anti-PD-L1 monoclonal antibody (Clone 22C3) and measured using a combined positive score (CPS), which is the number of PD-L1-positive cells (tumor cells, lymphocytes, and macrophages) as a percentage of the total number of tumor cells multiplied by 100. The MMR status was evaluated via IHC using monoclonal antibodies for anti-mutL homolog 1 (MLH1, ES05), anti-mutS homolog 2 (MSH2, FE11), anti-post-meiotic segregation increased 2 (PMS2, EP51), and anti-mutS homolog 6 (MSH6, EP49) (Agilent Technologies, Santa Clara, CA). Tumors lacking MLH1, MSH2, PMS2, or MSH6 expression were considered MMR-deficient (dMMR), whereas tumors that maintained MLH1, MSH2, PMS2, and MSH6 expressions were considered MMR-proficient (pMMR). Chromogenic *in situ* hybridization for Epstein–Barr virus (EBV)-encoded RNA (EBER) using fluorescein-labeled oligonucleotide probes (INFORM EBER Probe, Ventana) was carried out to assess the EBV status. All specimens in this study were reviewed by two pathologists (NS and TK).

### Assessments

Chemotherapy-related toxicity was assessed using the National Cancer Institute’s Common Terminology Criteria for Adverse Events (version 4.0). Surgical complications were graded according to the Clavien–Dindo classification (version 2.0). A computed tomography (CT) scan and an upper gastrointestinal endoscopy were carried out both before and after preoperative FLOT chemotherapy. Clinical and pathological stage was determined based on the 8th edition of the American Joint Committee on Cancer staging manual.^22^ Histopathologic effects were evaluated according to the Japanese classification of gastric cancer as follows: grade 3 (equivalent to pathological complete regression; no residual tumor cells); grade 2b (subtotal regression; residual tumor cells <10%); grade 2a (partial regression; tumor cells remain in >10% but <1/3); grade 1b (slight effect; tumor cells remain in >1/3 but <2/3); grade 1a (very slight effect; tumor cells occupy >2/3 of the tumorous area); and grade 0 (no evidence of effect).^23^^,^^24^ Grades 3 and 2b+3 correspond to pCR and major pathological response (MPR), respectively.

## Results

### Patient characteristics

The patient demographics are presented in Table 1. A total of 91 patients with resectable gastric cancer (60.4%), gastroesophageal junction cancer (34.1%), and esophageal adenocarcinoma (5.5%) were treated with perioperative FLOT therapy. The median age was 70 years (range: 29-82), and 78 patients (85.7%) had an ECOG PS of 0. At the time of diagnosis, 83 patients (91.2%) had T3 or higher-grade primary lesions, and 85 (93.4%) had lymph node metastasis. Before initiation of FLOT, staging laparoscopy was carried out in 28 (30.8%) of the 91 patients. Out of the 89 patients, 5 (5.5%) were HER2-positive, 5 out of 79 (5.5%) had dMMR tumors, and 3 out of 37 (3.3%) were EBV-positive.

**Table 1:** Baseline patient characteristics

Characteristics	(*n* = 91, %)
Gender	
Male	71 (78)
Female	20 (22)
Age (years) (median, range)	70 (29-82)
ECOG PS	
0	78 (86)
1	13 (14)
Tumor location	
Esophagus	5 (6)
Gastroesophageal junction	31 (34)
Gastric	55 (60)
Borrmann type	
Type 1	1 (1)
Type 2	18 (20)
Type 3	57 (63)
Type 4	10 (11)
Type 0	5 (6)
cT stage	
1b	3 (3)
2	5 (6)
3	45 (50)
4a	29 (32)
4b	9 (10)
cN stage	
N0	6 (7)
N1	49 (54)
N2	29 (32)
N3	7 (8)
Lauren’s type	
Diffuse	59 (65)
Intestinal	31 (34)
Not evaluable	1 (1)
HER2-positive^a^	5 (6)
EBV-positive^a^	3 (3)
MMR-deficient^a^	5 (6)
PD-L1 CPS (22C3)^a^	
<1	6 (7)
1-4	37 (41)
5-9	22 (24)
≥10	14 (15)

CPS, combined positive score; EBV, Epstein–Barr virus; ECOG, Eastern Cooperative Oncology Group; HER2, human epidermal growth factor receptor 2; MMR, mismatch repair; PD-L1, programmed death-ligand 1; PS, performance status.

a HER2, EBV, MMR, and PD-L1 CPS were analyzed in 82, 79, 79, and 79 cases, respectively.

### Safety and feasibility

**Figure 1:**
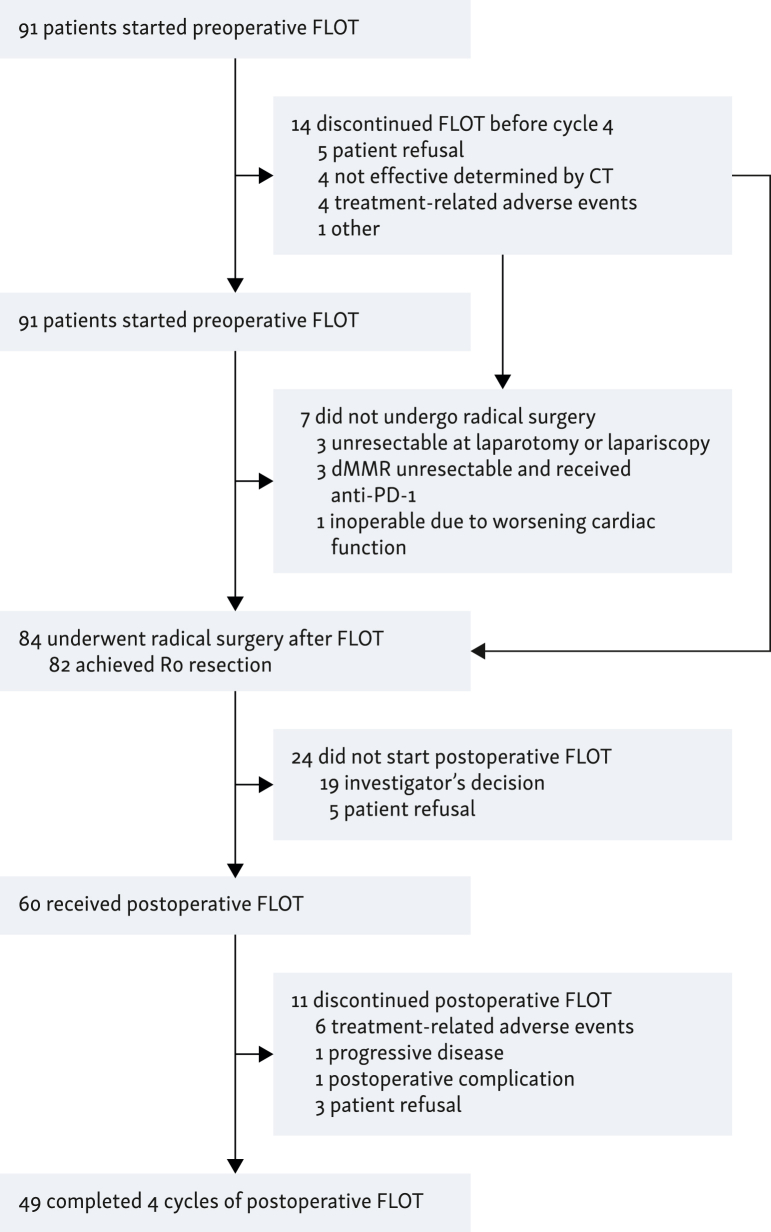
**Overview of patients receiving perioperative FLOT therapy**. This flowchart illustrates the treatment pathway of patients enrolled in this study. dMMR, mismatch repair-deficient; FLOT, 5-fluorouracil plus docetaxel plus oxaliplatin plus leucovorin; PD-1, Programmed cell death protein 1.

An overview of patients receiving perioperative FLOT therapy is presented in Figure 1. Out of the 91 patients, 77 (84.6%) completed four cycles of preoperative chemotherapy, whereas 14 (15.4%) discontinued FLOT before cycle 4 due to refusal in 5 patients, lack of efficacy determined via CT in 4, chemotherapy-related adverse events in 4 (2 with grade 3 hyperammonemic encephalopathy, 1 with grade 3 diarrhea, and another 1 with grade 2 mucositis), and other reasons in 1. Then, 84 out of 91 patients (92.3%) underwent radical surgery following FLOT. Among the remaining seven patients (7.7%), radical resection was not carried out due to various reasons: in three patients, peritoneal metastasis or adjacent organ invasion identified during laparotomy or laparoscopy; in another three patients with dMMR tumors, disease progression or vascular invasion observed in CT scans for restaging after FLOT; and in one patient, cardiac function deteriorated after FLOT rendering surgery infeasible. The first group of three patients subsequently received palliative chemotherapy. The second three patients with dMMR tumors were treated with anti-programmed cell death protein 1 (anti-PD-1) antibody, resulting in tumor shrinkage, and eventually, all three patients underwent R0 resection with pCR. After radical resection, 60 patients (65.9%) received at least one cycle of post-operative FLOT and 24 (26.4%) did not due to the investigator’s decision in 19 patients and refusal in 5. Furthermore, 49 patients (53.8%) completed four cycles of post-operative chemotherapy, whereas 11 (12.1%) discontinued FLOT due to chemotherapy-related adverse events in 6 patients (3 with grade 2/3 anorexia, 2 with grade 2 allergic reaction to oxaliplatin, and 1 with grade 2 pneumonitis), progressive disease in 1, post-operative complications in 1, and refusal in 3. Overall, 47 patients (51.6%) completed 8 cycles of perioperative FLOT treatment.

During the pre- and post-operative FLOT, all patients (100%) experienced any grade chemotherapy-related adverse events, with the most common events being leukopenia (56%), neutropenia (58.2%), anemia (96.7%), thrombocytopenia (63.7%), anorexia (62.6%), peripheral sensory neuropathy (47.3%), increased aspartate aminotransferase (AST) or alanine transaminase (ALT) (34.1%), and fatigue (30.8%) (Table 2). Chemotherapy-related adverse events of grade 3 or higher occurred in 60 patients (65.9%), including leukopenia (30.8%), neutropenia (50.5%), febrile neutropenia (5.5%), anorexia (7.7%), diarrhea (4.4%), increased AST or ALT (2.2%), hyperammonemic encephalopathy (3.3%), and allergic reaction to oxaliplatin (1.1%). The dose modifications and cumulative doses are presented in Supplementary Table S1, available at https://doi.org/10.1016/j.esmogo.2024.100050. Of the 91 patients, 59 (64.8%) had preoperative modifications, with 19 starting the first cycle of FLOT on a reduced dose and 39 (65.0%) of the 60 patients having post-operative modifications. The main adverse events that led to dose modifications were neutropenia, febrile neutropenia, and anorexia. Three patients discontinued oxaliplatin due to hypersensitive reaction. Patients aged 75 years and older had a significantly higher rate of dose reduction than those below the age of 75, both at the first cycle and at subsequent cycles (51.9% versus 7.8%; *P* < 0.0001, 77.8% versus 50.0%; *P* = 0.0141, respectively). Dose delays of >7 days occurred in 13 of 251 cycles (5.2%) during the preoperative period, and 23 of 159 cycles (14.5%) during the post-operative period, mainly due to neutropenia, febrile neutropenia, anorexia, and COVID-19 infection. In total, 80 patients (87.9%) received granulocyte colony-stimulating factor (G-CSF), including 54 patients (59.3%) who received them as primary prophylaxis. Pegylated G-CSF was administered on day 3 of the FLOT treatment to patients who received G-CSF prophylactically. The group that received primary prophylactic G-CSF showed significantly lower incidence of leukopenia and neutropenia, as well as fewer cases with dose delays of >7 days compared with the group that did not receive primary prophylactic G-CSF (Supplementary Table S2, available at https://doi.org/10.1016/j.esmogo.2024.100050).

**Table 2:** Chemotherapy-related adverse events

	Total (*n* = 91, %)		Neoadjuvant (*n* = 91, %)		Adjuvant (*n* = 60, %)	
	Any grades	Grade 3/4	Any grades	Grade 3/4	Any grades	Grade 3/4
All	91 (100)	60 (66)	91 (100)	53 (58)	59 (98)	26 (43)
Hematologic toxicity						
Leukopenia	51 (56)	28 (31)	47 (52)	23 (25)	20 (33)	7 (12)
Neutropenia	53 (58)	46 (51)	49 (53)	41 (45)	21 (35)	16 (27)
Anemia	88 (97)	4 (4)	79 (87)	2 (2)	55 (92)	3 (5)
Thrombocytopenia	58 (64)	2 (2)	47 (52)	2 (2)	34 (57)	0 (0)
Nonhematological toxicity						
Anorexia	57 (63)	7 (8)	43 (47)	3 (3)	28 (47)	4 (7)
Peripheral sensory neuropathy	43 (47)	0 (0)	33 (36)	0 (0)	22 (37)	0 (0)
AST or ALT increased	31 (34)	2 (2)	27 (30)	1 (1)	16 (27)	1 (2)
Fatigue	28 (31)	2 (2)	27 (30)	2 (2)	13 (22)	0 (0)
Diarrhea	25 (28)	4 (4)	20 (22)	3 (3)	11 (18)	1 (2)
Nausea	19 (21)	2 (2)	13 (14)	0 (0)	10 (17)	2 (3)
Mucositis	12 (13)	0 (0)	9 (10)	0 (0)	4 (7)	0 (0)
Allergic reaction to oxaliplatin	6 (7)	1 (1)	0 (0)	0 (0)	6 (10)	1 (2)
Febrile neutropenia	5 (6)	5 (6)	5 (6)	5 (6)	0 (0)	0 (0)
Hyperammonemic encephalopathy	3 (3)	3 (3)	3 (3)	3 (3)	0 (0)	0 (0)
Enteritis	3 (3)	0 (0)	2 (2)	0 (0)	1 (2)	0 (0)

ALT, alanine transaminase; AST, aspartate aminotransferase.

The surgical outcomes are presented in Supplementary Table S3, available at https://doi.org/10.1016/j.esmogo.2024.100050. Among the 84 patients who underwent radical surgery, 24 (28.6%) had post-operative complications of any grade. Post-operative complications of grade 3 or higher occurred in 11 patients (13.1%). Overall, no treatment-related deaths occurred.

### Efficacy

The histopathological findings are presented in Table 3. Among 84 patients who underwent radical surgery, 82 (97.6%) achieved R0 resection. One patient was classified as R1 resection due to positive peritoneal washing cytology, and another patient underwent R2 resection because of lymph node metastasis invading the recurrent nerve. Pathological T1 or lower was observed in 23 patients (27.4%), and pathological N0 was observed in 36 patients (42.9%). A total of 8 patients achieved grade 3, and 12 patients achieved grade 2b, resulting in a pCR rate of 9.5% (8 of 84 patients) and an MPR rate of 23.8% (20 of 84 patients). Seven of eight patients with primary tumor of grade 3 had ypN0. Two patients with dMMR tumors had grades 1a and 1b, respectively.

**Table 3:** Pathological response

	(*n* = 91, %)
R0	82 (90)
R1	1 (1)
R2	1 (1)
ypT stage^a^	
0	8 (9)
1a	6 (7)
1b	9 (10)
2	7 (8)
3	46 (51)
4a	7 (8)
4b	1 (1)
ypN stage^a^	
0	36 (40)
1	18 (20)
2	20 (22)
3a	7 (8)
3b	3 (3)
Histopathological effect^b^	
0	1 (1)
1a	24 (26)
1b	26 (29)
2a	13 (14)
2b	12 (13)
3	8 (9)

a Pathological stage was determined based on the American Joint Committee on Cancer staging manual (8th edition).

b Histopathological effects were evaluated according to the Japanese classification of gastric cancer. Grades 3 and 2b+3 correspond to pathological complete response (pCR) and major pathological response (MPR), respectively.

## Discussion

In this study, we retrospectively investigated the safety and efficacy of the perioperative FLOT regimen in patients with gastric, esophagogastric junction, or esophageal adenocarcinoma at our institution, demonstrating the acceptable feasibility of this treatment in Japanese clinical practice. To the best of our knowledge, this is the first report to show the clinical outcomes of FLOT therapy in this population.

In terms of safety and feasibility, 84.6% of patients completed four cycles of preoperative chemotherapy, and 92.3% of patients underwent radical surgery following FLOT, with 97.6% achieving R0 resection. Finally, 51.6% of patients completed eight cycles of perioperative FLOT treatment. These feasibilities were almost comparable to those in the FLOT4 trial.^9^ In this study, neutropenia was the most frequent chemotherapy-related adverse event, leading to dose modifications and delays. Although it has been reported that there is a significant difference in the incidence of docetaxel-induced severe neutropenia between Asian and non-Asian populations,^25^ the incidence of grade 3 or 4 neutropenia (50.5%) in this study, which involved only Japanese patients, was comparable to that in the FLOT4 trial (51%).^9^ This result can be attributed to the use of primary prophylactic G-CSF in approximately half of our patients. As demonstrated in Supplementary Table S2, available at https://doi.org/10.1016/j.esmogo.2024.100050, the incidence of grade 3 or 4 neutropenia was higher (80%) in patients who did not receive primary prophylactic G-CSF compared with those in the FLOT4 trial. In contrast, the group that received primary prophylactic G-CSF experienced a significantly lower incidence, with only two cases of grade 4 neutropenia or febrile neutropenia. These findings support a previous report that primary G-CSF prophylaxis reduces the incidence of neutropenia and treatment delays in patients receiving FLOT.^26^ Therefore, in high-risk patients, it may be worth considering prophylactic administration of G-CSF during FLOT treatment. In terms of surgical outcomes, only a few patients had serious surgical complications. Overall, no treatment-related deaths occurred.

Our preliminary efficacy results demonstrated a pCR rate of 9.5% and an MPR rate of 23.8%, which appear to be relatively lower than those in the FLOT4 study, with a pCR rate of 16% and an MPR rate of 37%.^9^ In comparison with FLOT4, more patients in our study had cT3 or higher and cN+ at diagnosis than in the FLOT4 trial, especially cT4 cases were remarkably higher in this study (42% versus 10%). In line with this observation, a randomized phase II study in China including only T4a or T4b disease also showed 20% MPR rate with FLOT.^27^ In the MATTERHORN trial, which included 19% Asian patients, the placebo plus FLOT group had an investigator-assessed pCR rate of 8%.^20^ Further investigation is needed to clarify whether these differences could happen by chance or be explained by higher T stage or different sensitivity to chemotherapy in Asian and non-Asian patients. In this study, 20.9% of patients started the first preoperative FLOT cycle with a reduced dose. Notably, the median age was 70 in our cohort, which was higher than that in the FLOT4 study, where only 24% of patients were 70 or older. This might be a major reason of higher dose modification in our study than the FLOT4 study.

In our patient cohort, 5.5% of patients had dMMR tumors, which was almost comparable to previous reports on localized gastric cancer.^28^, ^29^, ^30^ A meta-analysis of four randomized trials (MAGIC, CLASSIC, ARTIST, and ITACA-S) revealed that perioperative cytotoxic chemotherapy had no effect on survival in patients with microsatellite instability-high (MSI-H) locally advanced gastroesophageal cancer.^30^ In contrast, the phase II portion of the DANTE trial showed that 27% of patients with MSI-H/dMMR in the FLOT alone arm achieved MPR, suggesting a degree of effectiveness. In our study, which included five patients with dMMR tumors, three were deemed unresectable based on CT evaluation after FLOT, and the remaining two patients showed only minor effects in the resected specimens, with pathological findings categorized as grade 1a and 1b, respectively. The former three patients achieved tumor shrinkage after subsequent anti-PD-1 antibody therapy, leading to R0 resection with pCR. In a metastatic setting, a subgroup analysis of the CheckMate-649 study revealed that the magnitude of clinical benefit from nivolumab plus chemotherapy was greater in gastric cancer patients with MSI-H tumors.^31^ Furthermore, a subgroup analysis of the KEYNOTE-585 trial showed that patients with MSI-H tumors had better outcomes in terms of pCR rate, EFS, and overall survival when treated with the combination of chemotherapy and pembrolizumab.^19^ The MSI-H subgroup analysis results from the MATTERHORN trial are also highly anticipated to confirm the findings.

The major limitations of this study were its small sample size and retrospective, single-center design. Furthermore, several patients started the first cycle of preoperative chemotherapy with a reduced dose based on the physician’s discretion, which might affect the clinical outcomes in our patient cohort. Thus, we should also await information on the safety and efficacy of the FLOT regimen in a clinical trial involving Japanese patients, such as the MATTERHORN study, which compares FLOT plus durvalumab with placebo. Additionally, the evaluation of histopathologic effects in this study was based on the Japanese classification, and evaluations using the Becker classification or modified Ryan criteria as used in KEYNOTE585 or MATTERHORN were not carried out. It is important to note, however, that the definition of pCR or MPR (categories 1A and 1A+1B in the Becker classification) in either assessment are almost similar.

In conclusion, our study found that the perioperative FLOT regimen was well tolerated by Japanese patients with gastric, esophagogastric junction, or esophageal adenocarcinoma, with adequate dose modification. If the MATTERHORN trial involving the Japanese population meets its primary endpoints, perioperative FLOT combined with ICIs may become the standard of care. Our findings may be useful in establishing a FLOT regimen in the future.
