# Supplementary material for: Safety and efficacy of perioperative FLOT regimen in Japanese patients with gastric, esophagogastric junction, or esophageal adenocarcinoma: a single-institution experience
Source: ESMO Gastrointest Oncol. 2024 Apr 17;4:100050. doi: 10.1016/j.esmogo.2024.100050 (PMC12836703; doi:10.1016/j.esmogo.2024.100050)
Supplement: Supplementary data [file mmc1.docx]

**Supplemental tables of**

**Safety and efficacy of perioperative FLOT regimen in Japanese patients with gastric, esophagogastric junction, or esophageal adenocarcinoma: A single-institution experience.**

**Supplemental Table 1.** Chemotherapy administration

|  | Neoadjuvant  (n = 91, %) | Adjuvant  (n = 60, %) |
| --- | --- | --- |
| **Modification** | 59 (65) | 39 (65) |
| Modified from C1 | 19 (21) | 17 (28) |
| Modification reason (duplication+) |  |  |
| Investigator’s decision | 19 (21) | 2 (3) |
| AST or ALT increased | - | 2 (3) |
| Anorexia | - | 2 (3) |
| Peripheral neuropathy | - | 9 (15) |
| Others | - | 3 (5) |
| Modified after C2 | 53 (58) | 28 (47) |
| Modification reason (duplication+) |  |  |
| Neutropenia | 18 (20) | 5 (8) |
| Febrile neutropenia | 4 (4) | - |
| Anorexia | 16 (18) | 11 (18) |
| Fatigue | 7 (8) | 2 (3) |
| Diarrhea | 6 (7) | 2 (3) |
| Nausea | 1 (1) | 3 (5) |
| Allergic reaction to oxaliplatin | - | 3 (5) |
| Others | 11 (12) | 6 (10) |
| **Median dose percentage (median, range)** | 80 (40–100) | 80 (50–100) |
| **Dose delays >7days (cases/cycles)** | 11/13 (12/5) | 17/23 (28/15) |
| Dose delay reason |  |  |
| Neutropenia | 5/5 (6/2) | 4/6 (7/4) |
| Febrile neutropenia | 1/1 (1/0.4) | 0/0 (0/0) |
| Anorexia | 1/1 (1/0.4) | 2/2 (3/1) |
| COVID-19 | 2/2 (2/1) | 4/4 (7/3) |
| Others | 3/4 (3/2) | 11/12 (18/6) |
| **Cumulative dose (median, range)** |  |  |
| Docetaxel | 275 (65–400) | 230 (0–340) |
| Oxaliplatin | 460 (100–680) | 360 (0–580) |
| Fluorouracil | 14485 (3250–20800) | 12043 (2500–18000) |

Abbreviations: C1, first cycle; C2, second cycle; AST, aspartate aminotransferase; ALT, alanine transaminase

**Supplemental Table 2.** Comparative analysis of chemotherapy administration and adverse events with and without primary prophylactic granulocyte colony stimulating factor (GCSF).

|  | Neoadjuvant | | |  | Adjuvant | | |
| --- | --- | --- | --- | --- | --- | --- | --- |
|  | No GCSF  prophylaxis  (n = 45, %) | GCSF  prophylaxis  (n = 46, %) | p |  | No GCSF  prophylaxis  (n = 22, %) | GCSF  prophylaxis  (n = 38, %) | p |
| **Complete 4 cycles** | 35 (78) | 42 (91) | 0.074 |  | 16 (73) | 33 (87) | 0.173 |
| **Modification** | 29 (64) | 30 (65) | 0.939 |  | 14 (64) | 25 (66) | 0.866 |
| Modified from C1 | 10 (22) | 9 (20) | 0.755 |  | 7 (32) | 10 (26) | 0.649 |
| Modified after C2 | 27 (60) | 26 (57) | 0.737 |  | 10 (45) | 18 (47) | 0.886 |
| **Median dose percentage**  **(median, range)** | 80  (40-100) | 80  (50-100) | 0.791 |  | 67.5  (60-100) | 80  (50-100) | 0.780 |
| **Dose delays >7days**  **(cases)** | 9 (20) | 2 (4) | 0.002 |  | 11 (50) | 6 (16) | 0.005 |
| **AE (grade 3/4)*** | 38 (84) | 15 (33) | <0.001 |  | 19 (86) | 7 (18) | <0.001 |
| Leukopenia | 20 (44) | 3 (7) | <0.001 |  | 4 (18) | 0 (0) | <0.001 |
| Neutropenia | 36 (80) | 5 (11) | <0.001 |  | 15 (68) | 1 (3) | <0.001 |
| Febrile neutropenia | 4 (9) | 1 (2) | 0.160 |  | 0 (0) | 0 (0) | - |

*AE grades evaluated according to the Common Terminology Criteria for Adverse Events (version 4.0).

Abbreviations: C1, first cycle; C2, second cycle; AE, adverse event; GCSF, granulocyte colony stimulating factor

**Supplemental Table 3.** Surgical outcomes

|  | (n = 84, %) |  |
| --- | --- | --- |
| **Procedure** |  |  |
| Transthoracic subtotal esophagectomy | 31 (37) |  |
| Proximal gastrectomy with lower esophagectomy | 6 (7) |  |
| Total gastrectomy | 19 (19) |  |
| Distal gastrectomy | 31 (37) |  |
| **Approach** |  |  |
| Open surgery | 11 (13) |  |
| Minimally Invasive Surgery  (robotic, laparoscopic, thoracoscopic) | 73 (87) |  |
| **Median number of lymph nodes removed (median, range)** | 52.5 (20-123) |  |
| **Complications*** | Any grade | Grade 3/4 |
| Any | 24 (29) | 11 (13) |
| Intra-abdominal abscess | 4 (5) | 2 (2) |
| Pneumonia | 5 (6) | 1 (1) |
| Atelectasis | 2 (2) | 1 (1) |
| Lymph leakage | 3 (4) | 2 (2) |
| Anastomotic leakage | 2 (2) | 2 (2) |
| Non-occlusive mesenteric ischemia | 1 (1) | 1 (1) |
| Recurrent laryngeal nerve paralysis | 9 (11) | 0 (0) |
| **In-hospital death** | 0 (0) |  |

*Complication grades evaluated according to the Clavien–Dindo classification (version 2.0).
